# Treatment of Renal Cell Carcinoma with 2-Stage Total *en bloc* Spondylectomy after Marked Response to Molecular Target Drugs

**DOI:** 10.1155/2013/916501

**Published:** 2013-07-17

**Authors:** Yasuhiro Inoue, Hiroshi Takahashi, Yuichiro Yokoyama, Yasuaki Iida, Katsunori Fukutake, Ryo Takamatsu, Kazumasa Nakamura, Akihito Wada

**Affiliations:** Department of Orthopaedic Surgery, Toho University School of Medicine, 6-11-1 Omori-nishi, Ota-ku, Tokyo-8541, Japan

## Abstract

Metastatic renal cell carcinoma of the bone occurs at a high rate, and the prognosis is poor. In general, total *en bloc* spondylectomy is considered when there is only one vertebral metastasis and the primary disease is treated. However, palliative surgery is selected when the primary disease is not being treated or metastasis occurs to an important organ. We encountered a patient in whom lung and vertebra metastases were already present at the time of the first examination at our department and the prognosis was considered poor. However, molecular targeted therapy was markedly effective and enabled 2-stage total *en bloc* spondylectomy. As of one year after total *en bloc* spondylectomy, the condition has improved to cane gait, and surgery for lung metastasis is planned. Molecular target drugs might markedly change the current therapeutic strategy for renal cell carcinoma.

## 1. Introduction 

 Treatment of metastatic renal cell carcinoma of the spine includes chemotherapy, radiotherapy, and surgical resection. While the therapeutic strategy is changing with the appearance of molecular target drugs, the prognosis of patients with this disease remains generally poor. We encountered a patient with metastatic renal cell carcinoma of the spine for whom 2-stage total *en bloc* spondylectomy was able to be performed because the molecular target drugs were markedly effective.

## 2. Case

The patient is a 49-year-old male; and his chief complaint is gait disturbance with a past medical history of duodenal ulcer.

*History of the Present Illness*. The patient experienced right abdominal pain and was diagnosed as having a duodenal ulcer by a physician 4 years before. During further examination and treatment, a 4 cm mass was found in the right kidney on abdominal CT. The mass was diagnosed as a right renal cancer at the urology department of our hospital and was treated with nephrectomy. The histopathological diagnosis was clear cell carcinoma. Upon examination of the whole body, metastases were noted in the right lung and Th11 vertebra, and radiotherapy was initiated for these metastatic lesions. Treatment with a molecular target drug, Sorafenib, was initiated one month later, but both pulmonary and spinal lesions remained progressive. Thus, the drug was switched to another molecular target drug, Sunitinib, 2 months later. The progression of the metastatic lesions was prevented, but spinal deformity progressed slowly, and numbness had appeared in the bilateral lower limbs 1.5 years before. The patient gradually became unable to walk about one year before, and was referred to our department.

*Status upon Admission*. In the spine, kyphosis was noted with a vertex at Th11. The patellar and Achilles tendon reflexes were enhanced bilaterally. Hypesthesia was noted in the region innervated by Th12 and below, and weakness of the iliopsoas and lower muscles was noted on manual muscle testing (MMT). No bladder or rectal disturbances were noted. 

**Figure 1:**
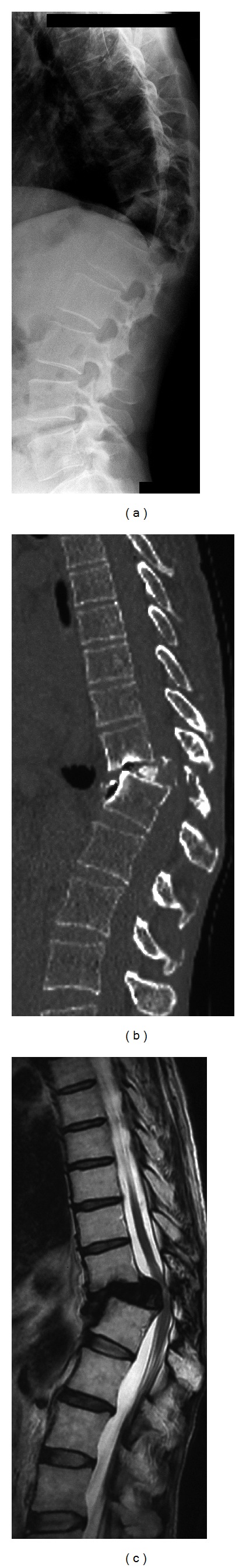
Images of the lumbar vertebrae on the first examination. (a) Plain X-ray radiography. (b) CT (MPR). (c) T2-weighted MRI.

*Imaging Findings*. On plain X-ray radiography, crushed Th11 and anterior dislocation of Th10 were noted. On MRI, severe compression of the spinal cord was noted at Th11 (Figure 1). A metastatic lesion was noted in the right apical pulmonary region on chest CT, but no metastasis was noted in any other organ on abdominal CT or bone scintigraphy. 

**Figure 2:**
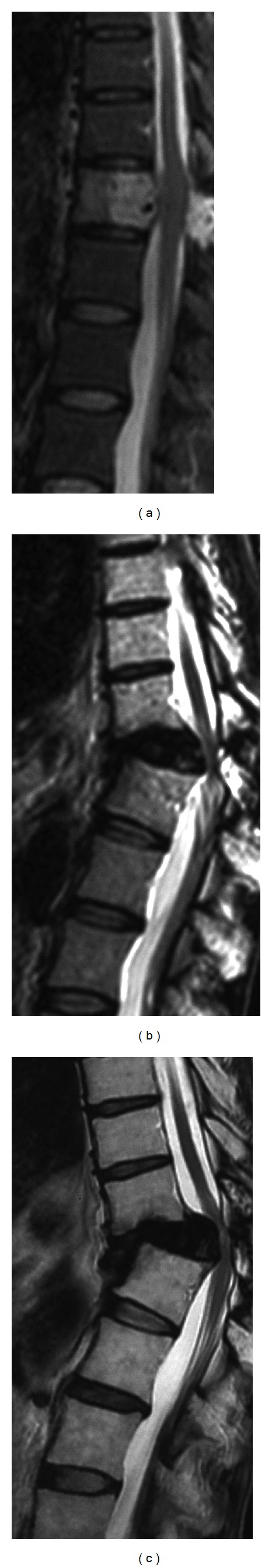
Time-course changes on T2-weighted MRI. (a) On the first examination at the urology department. (b) After one year. (c) After 3 years.

The time-course changes in spinal MRI from the first examination at the urology department to the first examination at our department are shown in Figure 2. Since the deformation progressed slowly, severe kyphosis was noted on the first examination at our department, but the progression of paralysis was relatively slow. The metastatic lesion was present only in a single vertebra, but palliative surgery for posterior decompression and fixation was planned because lung metastasis was already present.

**Figure 3:**
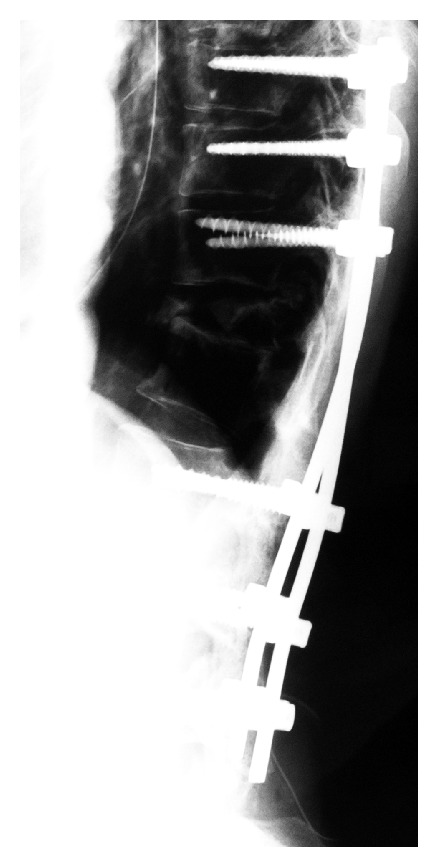
Lateral view on plain X-ray radiography after the first surgery.

*Surgical Findings*. Th11 laminectomy was performed, the bone metastatic region was resected concomitantly, as much as possible, and posterior fixation of Th8-L3 was applied (Figure 3). Regarding the bone quality, the hardness of the vertebral arch had increased, suggesting that the molecular target drugs were effective. The bone metastatic region was present in the Th11 transverse process over the lateral side of the vertebral arch, and this region was examined thoroughly. In the dural tube, the yellow ligament and membranous scar tissue were adhered to the dura. These were dissected as much as possible. When resection was advanced to the root of the Th11 vertebral arch, potential was improved on electrospinographic monitoring.

**Figure 4:**
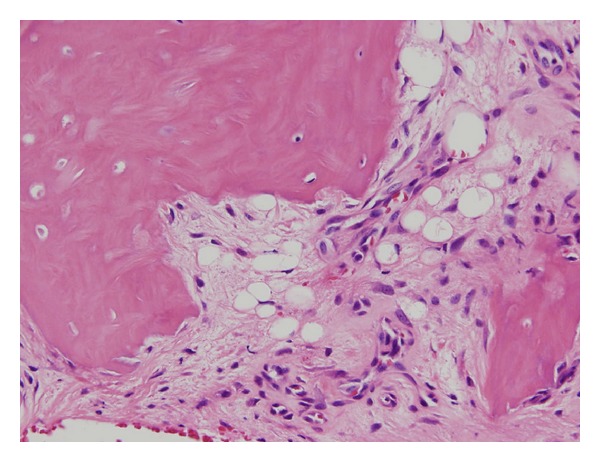
Histopathological findings, HE staining (×40).

*Pathological Findings.* In the excised specimen, the bone tissue was partially accompanied by fibrosis, and no tumor cells were present (Figure 4).

**Figure 5:**
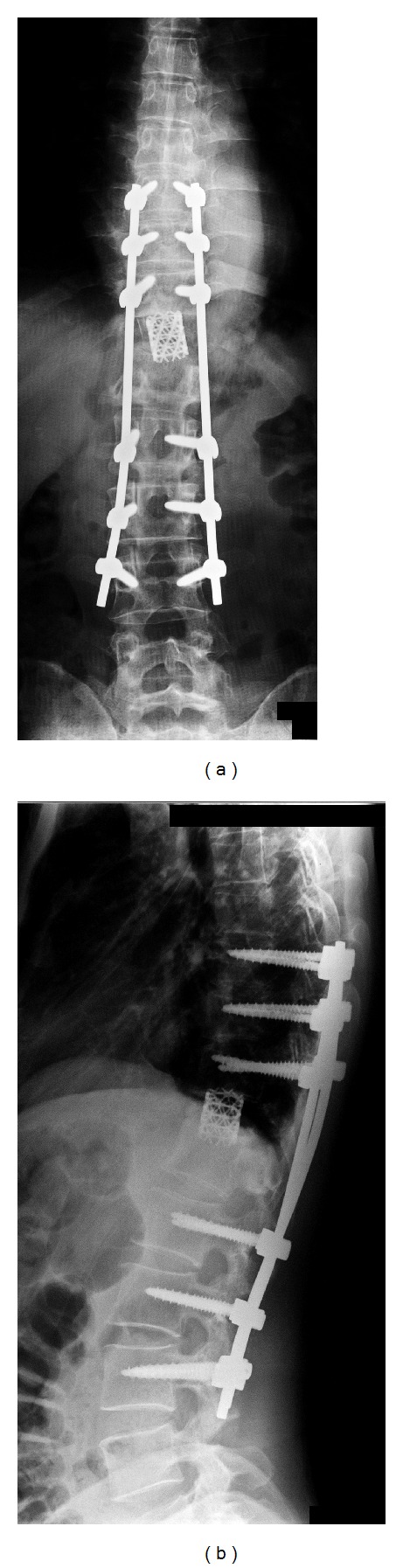
Images after the second surgery, HE staining (×40). (a) Frontal view on plain X-ray radiography. (b) Leteral view on plain X-ray radiography.

*Course*. Kyphosis was corrected by the first surgery. Since a defect was evident in the anterior column, no tumor cells were observed on pathological examination, and the pulmonary lesion did not progress; total *en bloc* spondylectomy was performed 2 weeks after the first surgery. Total *en bloc* spondylectomy of Th11 was performed by left thoracotomy due to the presence of a crushed Th11 bone fragment on the left side, followed by anterior fixation of Th10–12 with a cage containing autologous bone (Figure 5). On pathological examination of the excised vertebra, bone tissue was accompanied by necrosis and degeneration, and there was no sign of the metastatic renal cell carcinoma. As of one year after surgery, the condition has improved to cane gait. Since no progression of the right metastatic lung tumor has been noted on CT, endoscopic excision of the malignant pulmonary tumor is planned.

## 3. Discussion 

 Bone metastasis following renal cell carcinoma has been noted in about 30% of patients, which is the fourth highest incidence following prostate (50–70%), breast (30–70%), and lung cancers (30–65%). The 5-year survival rate has been reported to be 10–75% [1].

Osteolysis can be observed on plain X-ray radiography and may cause pathological fracture. Since chemotherapy, radiotherapy, or immunotherapy are not generally expected to be effective for bone metastatic lesions, surgical treatment is selected, when applicable [2]. When surgical treatment is selected, in general, the following conditions are favorable prognostic factors: renal cell carcinoma-derived tumor thrombus is absent in the inferior vena cava, bone metastasis is diagnosed more than 2 years after the diagnosis of renal cell carcinoma, extraosseous metastasis is absent, and primary disease is treated [3] (Table 1). In our patient, vertebral and lung metastases were already present upon the first examination, suggesting a poor prognosis. Surgical procedures for metastatic spinal tumor are roughly divided into 2 types: excisional surgery in which the tumorous vertebra is resected, processed by curettage, and reconstructed with an artificial vertebra or bone cement and palliative surgery in which pain is relived and paralysis is improved by posterior decompression and reconstruction of the spinal support [4]. Excisional surgery is selected for patients with single vertebral metastasis and favorable general condition, and palliative surgery is selected for those with metastasis of multiple vertebrae and a poor general condition. In our patient, molecular targeted therapy was markedly effective and enabled 2-stage excisional surgery of total *en bloc* spondylectomy. Reportedly, antivascularization molecular target drugs have been reported to improve oxygenation in tumors by normalizing abnormal tumor blood vessels, which strengthens the antitumor effect of radiation. Reformation of the therapeutic strategy for metastatic renal cell carcinoma of the bone by molecular target drugs is expected [5].

## 4. Conclusion

The prognosis of metastatic renal cell carcinoma of the spine is generally poor, but we consider that active surgery is an option for patients for whom molecular targeted therapy is effective. 

## Figures and Tables

**Table 1:** Prognostic factors of renal cell carcinoma.

Factor	No.	Median survival (mo)	*P* value
Tumor thrombus in primary tumor			
Presence	26	5	
Absence	18	19	0.0246
Unknown	5		
Interval between diagnosis of RCC and bone metastasis			
<24 mo	34	8	
>24 mo	16	34	0.0155
Extraosseous metastasis			
Presence	29	8	
Absence	21	33	0.0084
Local treatment (surgery and/radiation)			
Yes	29	17	
No	21	8	0.0348

*RCC: renal cell carcinoma [3].

## References

[1] Zekri J, Ahmed N, Coleman RE, Hancock BW (2001). The skeletal metastatic complications of renal cell carcinoma. *International Journal of Oncology*.

[2] Kollender Y, Bickels J, Price WM (2000). Metastatic renal cell carcinoma of bone: indications and technique of surgical intervention. *Journal of Urology*.

[3] Toyoda Y, Shinohara N, Harabayashi T (2007). Survival and prognostic classification of patients with metastatic renal cell carcinoma of bone. *European Urology*.

[4] Tokuhashi Y, Ajiro Y, Umezawa N (2009). Outcome of treatment for spinal metastases using scoring system for preoperative evaluation of prognosis. *Spine*.

[5] Winkler F, Kozin SV, Tong RT (2004). Kinetics of vascular normalization by VEGFR2 blockade governs brain tumor response to radiation: role of oxygenation, angiopoietin-1, and matrix metalloproteinases. *Cancer Cell*.

